# Anatomic Correlation of the Mini-Mental State Examination: A Voxel-Based Morphometric Study in Older Adults

**DOI:** 10.1371/journal.pone.0162889

**Published:** 2016-10-14

**Authors:** Mickael Dinomais, Sebastien Celle, Guillaume T. Duval, Frederic Roche, Samir Henni, Robert Bartha, Olivier Beauchet, Cedric Annweiler

**Affiliations:** 1 Université d’Angers, Laboratoire Angevin de Recherche en Ingénierie des Systèmes (LARIS)–EA7315 F-49000 France, LUNAM, Université d’Angers, Département de Médecine Physique et de Réadaptation, CHU Angers, F-49933, France; 2 Service de Physiologie Clinique et de l’Exercice, CHU, Saint-Etienne, France, EA 4607 "SNA EPIS" Faculté de Médecine J Lisfranc UJM, PRES Université de Lyon, F-42023, France; 3 Department of Neuroscience, Division of Geriatric Medicine and Memory Clinic, Angers University Hospital, UPRES EA 4638, University of Angers, UNAM, Angers, France; 4 Department of Sports Medicine and Vascular Investigations, University Hospital, Angers, France; 5 Robarts Research Institute, Department of Medical Biophysics, Schulich School of Medicine and Dentistry, the University of Western Ontario, London, Ontario, Canada; 6 Department of Medicine, Division of Geriatric Medicine, Sir Mortimer B. Davis—Jewish General Hospital and Lady Davis Institute for Medical Research, McGill University, Montreal, Quebec, Canada; Indiana University Bloomington, UNITED STATES

## Abstract

The clinical utility of the Mini-Mental State Examination (MMSE) and its shorter version (SMMSE) is still debated. There is a need to better understand the neuroanatomical correlates of these cognitive tests. The objective of this cross-sectional study was to determine whether lower MMSE and SMMSE scores correlated with focal brain volume reduction in older adults. Participants from the GAIT study (n = 207; mean, 70.9±5.9 years; 57% female; mean MMSE 26.2±3.9; mean SMMSE 5.1±1.1) were evaluated using the MMSE and SMMSE and received a 1.5-Tesla MRI scan of the brain. Cortical gray and white matter subvolumes were automatically segmented using Statistical Parametric Mapping. Age, gender, education level, and total intracranial volume were included as potential confounders. We found correlations between the MMSE score and specific cortical regions of the limbic system including the hippocampus, amygdala, cingulate gyrus, and parahippocampal gyrus, independently of the diagnostic category (i.e., mild cognitive impairment or Alzheimer disease or controls). Regarding correlations with the SMMSE score, only one cluster in the left hippocampus was identified, which overlapped with the cluster that was positively correlated with the MMSE score. There were no correlations with the volume of white matter. In conclusion, worse MMSE and SMMSE scores were associated with gray matter atrophy mainly in the limbic system. This finding highlights that atrophy of specific brain regions are related to performance on the MMSE and the SMMSE tests, and provides new insights into the cognitive function probed by these tests.

## Introduction

The Mini-Mental State Examination (MMSE), published in 1975 by Folstein and colleagues, is a practical method of grading cognitive impairment [1,2] that has gradually become the most common cognitive test, used by almost 9 out of 10 specialists [3]. The MMSE is typically applied to help evaluate cognitive performance as a whole [1] and detect suspected dementia [4]. In addition to diagnosis, it has been used extensively to grade cognitive impairment in trials and observational studies of dementia [4,5]. Despite its widespread use, many non-specialists consider the MMSE too time consuming to administer [6], and a shorter version of the MMSE (i.e. short MMSE, SMMSE) was recently proposed [7]. The metrological properties of the latter version were very similar to those of the original MMSE [8], making it an interesting alternative, but the same concerns about diagnostic accuracy exist for it as for the MMSE.

More than 70 validation studies of the MMSE are available to date [5]. Although such a large evidence base enhances the understanding and knowledge of the utility of the MMSE by health professionals, opinion remains divided about its accuracy, relevance, and reliability [4,5]. One reason is that most validation studies were underpowered and hence may have given a misleading impression of accuracy [9]. Additionally, two recent meta-analyses of 34 dementia studies reported that the MMSE actually offers only modest accuracy in diagnosing cognitive disorders [5], and should be used mainly in low prevalence settings [4].

As the clinical utility of the MMSE and SMMSE scores is still debated, there is a need to better understand how these cognitive measures relate to changes in brain structure. Surprisingly, despite the widespread use of the MMSE in clinical and research settings, very few studies have examined the relationship between these cognitive tests and brain structure measured by imaging [10–13]. Reporting an anatomical correlation with test performance would strengthen their reliability and interest in assessing brain function. We had the opportunity to examine the correlations of MMSE and SMMSE scores with gray matter (GM) and white matter (WM) volumes in a large representative community survey of older adults with memory complaint in the GAIT (Gait and Alzheimer Interactions Tracking) study [14]. The objective of this cross-sectional analysis was to determine whether lower MMSE and SMMSE scores correlated with focal brain volume reduction.

## Materials and Methods

### Participants

We studied participants with subjective memory complaint followed in the Memory Clinic of Angers University Hospital, France, and recruited in the GAIT study between November 2009 and January 2013. The GAIT study is an observational cross-sectional study designed to examine gait in older community-dwellers reporting subjective memory complaint. The sampling and data collection procedures have been described elsewhere [14]. The main exclusion criteria were age below 60 years, MMSE score <10, inability to walk independently, history of stroke, any acute medical illness in the preceding 3 months, current delirium, severe depression, and inability to understand or answer the study questionnaires. All participants included in the present analysis received a full medical examination, a neuropsychological assessment, and a 1.5 Tesla magnetic resonance imaging (MRI) scan of the brain.

### MMSE and SMMSE scores

Neuropsychological assessment was performed during a face-to-face examination performed by a neuropsychologist at the time of the MRI scan. The MMSE is composed of 19 individual tests of 11 domains covering orientation, learning, attention or calculation (serial sevens or spelling), recall, naming, repetition, comprehension (verbal and written), writing, and construction [1]. The MMSE score ranges from 0 to 30 (best). The SMMSE score was calculated, as previously published, from 6 memory items of the MMSE following the formula: [free recall of 3 words + delayed recall of 3 words] [7]. The SMMSE score ranges from 0 to 6 (best). Here, the SMMSE was not performed as a single test, but built from the MMSE.

### MRI procedure

#### MRI acquisition

All images were acquired on the same 1.5 Tesla MRI scanner (Magnetom Avanto, Siemens Medical Solutions, Erlangen, Germany) at the University Hospital of Angers, France, using a standard MRI protocol [15]. A high-resolution 3D T1-weighted volume was obtained covering the whole brain (acquisition matrix = 256x256x144, FOV = 240mm, TE/TR/TI = 4.07ms/2170ms/1100ms, flip angle = 15°, voxel size 1mm×1mm×1.3mm).

#### Voxel-based morphometry with DARTEL analysis

Basic voxel-based morphometry (VBM) with DARTEL analysis (http://www.neuro.uni-jena.de/vbm8/VBM8-Manual.pdf) was conducted using standard functionalities (default options) available in the VBM8 toolbox (http://dbm.neuro.uni-jena.de/VBM8/) implemented in the SPM8 software (http://www.fil.ion.ucl.ac.uk/spm). VBM analysis was performed following standard procedures (http://www.fil.ion.ucl.ac.uk/~john/misc/VBMclass10.pdf). The default options of the VBM procedure provided in VBM8 were used. Native MR images were segmented into distinct tissue classes: GM, WM and cerebrospinal fluid (CSF), using a new segmentation approach available in SPM8. The extended option “thorough cleanup”, which is particularly useful for atrophic brain, was used during the first module “estimate and write”. Customized DARTEL-templates were created using affine registered tissue segments [16]. These customized DARTEL templates replaced the default DARTEL templates. Hence, GM and WM volumes were normalized using high dimensional spatial normalization to a customized DARTEL template. A modulation of the segmented and normalized GM (modulated GM) and WM (modulated WM) volumes were undertaken [17]. The final resolution of the modulated GM and WM images was 1.5mm×1.5mm×1.5mm, but these were smoothed with a 6 mm FWHM (full-width-at-half-maximum) Gaussian Kernel to minimize individual gyral variations. All images were visually inspected to ensure that the steps described above were successful and that each modulated GM and WM map covered the whole brain.

### Covariables

The cognitive diagnosis was made during multidisciplinary meetings involving geriatricians, neurologists and neuropsychologists of Angers University Memory Center, France, and was based on a variety of standardized neuropsychological tests, physical examination findings, blood tests and MRI brain imaging [14]. Clinical suspicion of dementia was diagnosed using the Diagnostic and Statistical Manual of Mental Disorders, fourth edition, criteria [18]. Probable Alzheimer disease (AD) was diagnosed according to the criteria of the National Institute of Neurological and Communicative Disorders/Alzheimer’s Disease and Related Disorders Association working group [19]. Mild Cognitive Impairment (MCI) was diagnosed according to Dubois et al. consensus criteria [20]. Nondemented participants without MCI and who had normal neuropsychological and functional performance were considered as cognitively healthy individuals (CHI).

The following variables were used as potential confounders in the analyses: age, gender, education level, and total intracranial volume (TIV). Education level was evaluated using standardized questionnaires. Higher education level was defined as an education level (whether undergraduate or postgraduate) following the completion of a school providing a secondary education. The TIV was approximated for each participant by calculating the sum of GM, WM and CSF maps obtained during the pre-processing steps.

### Ethics

The study was conducted in accordance with the ethical standards set forth in the Helsinki Declaration (1983). Written informed consent was obtained at enrolment and the entire study protocol was approved by the University of Angers Ethical Review Committee (CPP Ouest II—2009–12).

### Data analysis

The smoothed, modulated, normalized imaging datasets were used for voxelwise statistical analysis using SPM8. We correlated GM and WM volumes with the MMSE score and the SMMSE score (quantitative variables) using separate multiple regressions models. All statistical parametric maps were interpreted after applying a family-wise error (FWE) correction for multiple comparisons at the whole-brain level with a significance level p-value (corrected) < 0.05. Minimum cluster size was set at 10 contiguous voxels. Anatomy toolbox 2.1 was used for anatomical localizations [21].

To facilitate interpretation and for the sake of visual clarity, correlation scatter plots were built for the voxel that showed within the cluster the highest correlation with the MMSE and SMMSE scores in the VBM analyses (i.e., local maximum peak voxel). Because the brain processing of cognitive functions generally involves symmetrical left and right hemispheres to a different degree, we built the symmetrical counterpart of the local maximum peak voxel to allowing visual exploratory comparison of the magnitude of correlations in each homologous region on each side (right and left). In addition, scatter plots were stratified by disease categories (i.e., CHI, MCI, or AD) to give visual overwiew of the relationships between the brain subvolumes and the MMSE or SMMSE scores according to the three diagnostic groups,

## Results

Two hundred and seven participants (mean±standard deviation 70.88±5.88 years; 57% female; 22% graduate studies; 33.8% diagnosed as cognitively healthy; 44.9% with MCI; 21.2% with AD) met the selection criteria and were included in the present analysis. The mean MMSE score was 26.2±3.9 [95% confidence interval (CI): 25.7–26.7] (range, 10–30) among studied participants, and the mean SMMSE score was 5.1±1.1 [95%CI: 4.9–5.3] (range, 0–6). The mean TIV was 1397.0±127.3 cm^3^ [95%CI: 1379.0–1414.0] (range, 1089.0–1699.0).

**Fig 1:**
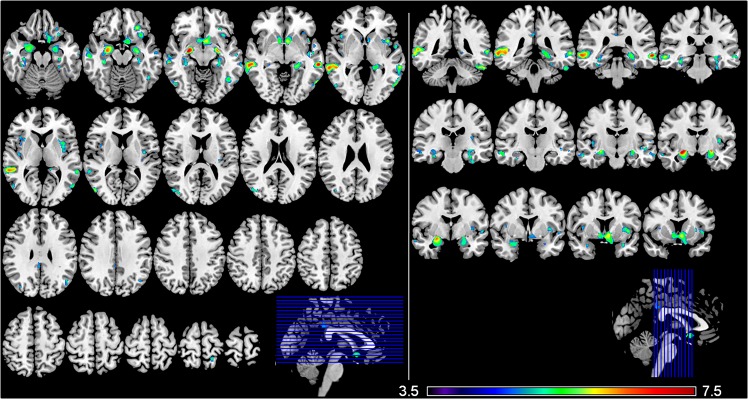
Gray matter regions showing a positive correlation with MMSE score. The statistical map is co-registered and superimposed on 3D-T1 (A) axial (MNI coordinates: z = -20 to z = +75) and (B) coronal slices (MNI coordinates: y = -42 to y = +12) (MNI T1 template available on MRICRON software). Results are showed with a significance of P<0.05 corrected for multiple comparisons (FWE-corrected). Colour bar indicates the *t*-score for the regression slopes (from blue colour [*t* = 3.5] to red colour [*t* = 7.5]).

**Fig 2:**
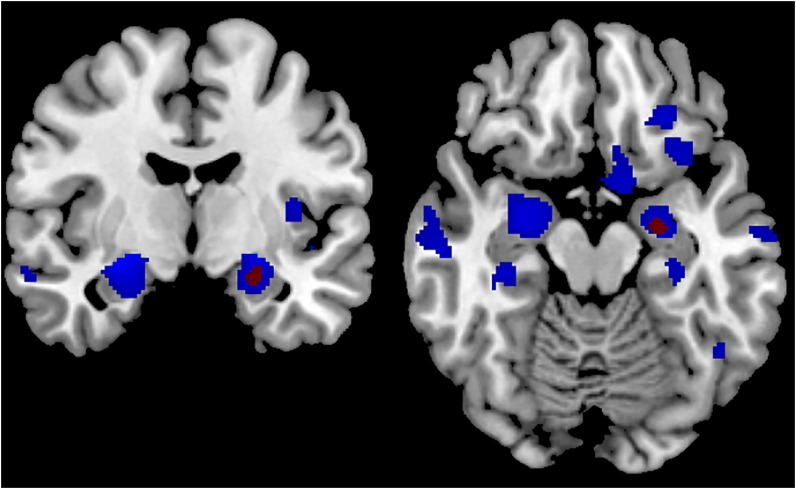
Gray matter regions showing a positive correlation with MMSE score (blue color) and SMMSE score (red color). The statistical map is co-registered and superimposed on 3D-T1-weighted axial (MNI coordinates y = -8) and coronal slices (MNI coordinates z = -16) (MNI T1-weighted template available on MRICRON software). Results are showed with a significance of P<0.05 corrected for multiple comparisons (FWE-corrected).

**Fig 3:**
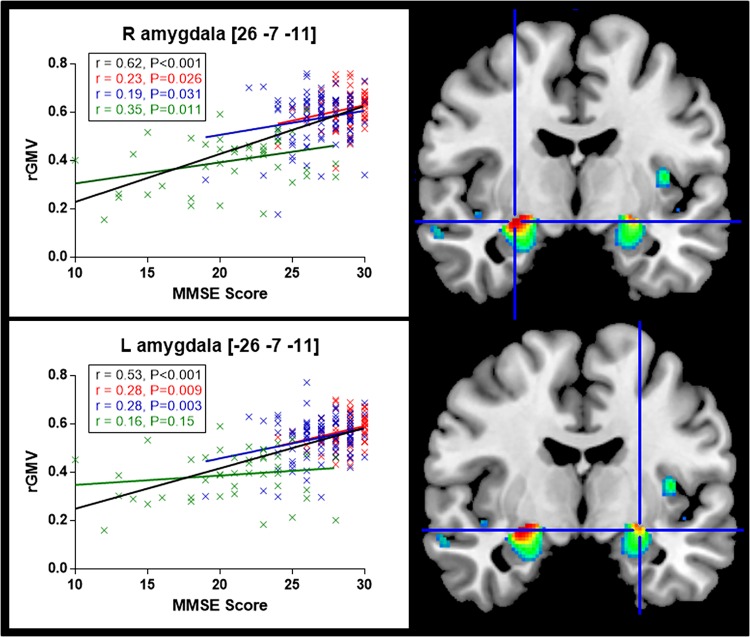
Correlation between the MMSE score and the regional gray matter volume (rGMV) in right (R) and left (L) amygdala according to participants’ diagnostic category. Plots points indicate the quantification of the VBM signal sampled from the voxels of the right and left amygdala. In black: whole sample of participants; red: cognitively healthy participants; blue: participants with MCI; green: participants with AD. L, left in [x y z], MNI coordinates. Results are shown for P<0.05 corrected for multiple comparisons FWE corrected.

Fig 1 illustrates the brain regions that positively correlated with the MMSE score after adjusting for age, gender, TIV, and education level. The VBM-DARTEL analysis identified large bilateral clusters overlapping cortical regions of the limbic system including notably hippocampus, amygdala, cingulate gyrus, and parahippocampal gyrus. Regions showing the highest correlation with the MMSE score were: the right amygdala (MNI coordinates [x y z]; [26–7–11], *t* = 7.26), the left middle temporal gyrus ([-62–33–5], *t* = 7.00), and the right middle temporal gyrus ([62–33–5], *t* = 6.62) (Table 1 provides detailed results on the clusters size and MNI coordinates). The correlations between the MMSE score and the regional GM volume in right and left amygdala (Fig 2) persisted while considering each disease category separately (i.e., CHI, MCI, and AD) (Fig 3), which confirmed the interest of the MMSE for this brain region in each subgroup of patients. In contrast, no significant negative correlation was found between the MMSE score and the GM volume across the whole brain.

**Table 1:** Detailed results of the VBM analysis: correlation of gray matter volume with MMSE score and SMMSE score.

	Number of voxels	Localization	MNI coordinates			*t-*score
			x	y	z	
Brain subvolume positively correlated with MMSE score						
Cluster 1	1646	R Middle Temporal G	62	-33	-5	6.62
			63	-37	-2	6.56
			60	-39	3	6.47
			59	-40	4	6.46
			51	-37	0	6.44
			60	-30	-9	6.39
			57	-15	-18	5.64
			59	-16	-17	5.61
			62	-6	-15	5.17
Cluster 2	1218	L Caudate Nucleus	-6	9	-8	6.11
		R Caudate Nucleus	9	9	-6	5.72
		L Rectal G	-11	18	-11	5.37
			-8	21	-18	5.19
		L Inferior Frontal G (p.Orbitalis)	-18	15	-20	5.05
**Cluster 3**	**1001**	**R Amygdala**	**26**	**-7**	**-11**	**7.26**
		R Temporal Pole	30	5	-24	5.46
Cluster 4	892	L Middle Temporal G	-62	-33	-5	7.00
			-62	-42	3	5.97
			-56	-67	1	5.79
			-60	-58	1	5.34
			-60	-46	-2	5.27
			-63	-51	0	5.07
		L Inferior Temporal G	-59	-63	-5	6.08
Cluster 5	786	L Hippocampus	-24	-34	-5	5.79
			-27	-31	-8	5.65
			-29	-22	-15	5.18
		L Fusiform G	-36	-22	-29	5.36
			-30	-27	-26	5.26
			-30	-37	-20	5.12
Cluster 6	579	L Temporal Pole	-33	9	-23	5.38
Cluster 7	481	L Inferior Frontal G (p.Orbitalis)	-27	33	-15	6.01
			-30	29	-12	5.88
			-33	20	-15	5.66
		L Insula Lobe	-29	21	-18	5.44
			-35	24	-3	4.91
Cluster 8	460	L Insula Lobe	-41	9	0	6.11
			-39	-7	9	5.50
Cluster 9	427	R Middle Temporal G	50	-75	10	5.85
			51	-69	1	5.46
		R Middle Occipital G	41	-78	16	5.58
			45	-75	18	5.39
			36	-76	27	5.35
Cluster 10	379	R ParaHippocampal G	33	-25	-17	5.78
			33	-19	-21	5.37
		R Fusiform G	36	-34	-20	5.54
			38	-31	-18	5.49
			30	-31	-21	4.97
Cluster 11	301	L Inferior Temporal G	-56	-40	-26	6.30
			-48	-42	-26	5.92
Cluster 12	215	L Inferior Temporal G	-45	-58	-9	5.50
Cluster 13	170	R Insula Lobe	41	18	1	5.45
			38	6	7	5.12
Cluster 14	116	L Superior Temporal G	-51	-12	-5	5.54
			-44	-12	-5	5.30
		L Middle Temporal G	-47	-16	-8	5.09
Cluster 15	109	L Middle Temporal G	-59	-15	-11	5.24
			-66	-12	-18	5.13
			-60	-10	-15	5.00
Cluster 16	93	L Angular G	-48	-64	28	5.29
			-44	-64	34	5.11
			-47	-70	30	4.99
Cluster 17	83	R Middle Cingulate Cortex	3	-37	31	5.09
		L Middle Cingulate Cortex	0	-39	39	4.82
Cluster 18	79	L Superior Temporal G	-54	0	-2	5.51
Cluster 19	77	R Inferior Temporal G	48	-67	-9	5.66
			54	-60	-9	4.85
Cluster 20	51	R Insula Lobe	42	-12	4	5.12
Cluster 21	46	L Superior Parietal Lobule	-21	-48	70	5.47
Cluster 22	27	L Insula Lobe	-35	-22	13	5.00
Cluster 23	26	L Superior Temporal G	-59	-10	7	5.05
Brain subvolume positively correlated with SMMSE score						
**Cluster 1**	**67**	**L Hippocampus**	**-24**	**-9**	**-17**	**5.25**
			-20	-3	-23	5.04

R: right; L: left; MNI: Montreal Neurological Institute. In bold characters: clusters with the highest *t*-score. See also Fig 1 and Fig 2.

**Fig 4:**
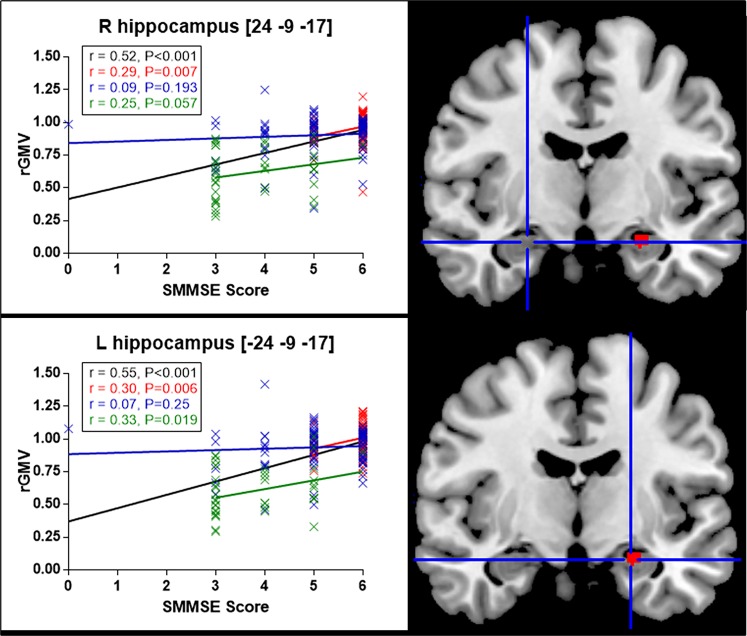
Correlation between the SMMSE score and the regional gray matter volume (rGMV) in right (R) and left (L) hippocampus according to participants’ diagnostic category. Plots points indicate the quantification of the VBM signal sampled from the voxels of the right and left hippocampus. In black: whole sample of participants; red: cognitively healthy participants; blue: participants with MCI; green: participants with AD. L, left in [x y z], MNI coordinates. Results are shown for P<0.05 corrected for multiple comparisons FWE corrected.

Regarding correlations with the SMMSE score, the VBM-DARTEL analysis identified only one cluster located in the left hippocampus ([-24–9–17], *t* = 5.25; [-20–3–23], *t* = 5.04), which overlapped with the cluster that was positively correlated with the MMSE score (Fig 2, Fig 4, and Table 1).

Of note, using a more permissive threshold in VBM, the patterns of brain structure where the GM volume positively correlated with the MMSE and SMMSE scores were the same but appeared more symmetrical (data not shown). However, the plot correlations between bilateral homologous regions showed a persistent right and left predominance for amygdala and hippocampus respectively (Fig 3 and Fig 4).

Finally, after adjusting for age, gender, TIV and education level, the VBM-DARTEL multiple regression analysis revealed that the MMSE and SMMSE scores did not correlate (positively or negatively) with the volume of WM across the whole brain, even when a more permissive approach was applied.

## Discussion

Our results show that, among community-dwelling older adults with memory complaint, the MMSE score correlated positively with GM volume in the limbic system and in the middle temporal gyrus. The SMMSE score correlated positively with GM volume only in the left hippocampus and associated structures. There was no correlation with the WM volume. These findings provide new insights into understanding which brain structures and therefore functions are actually probed by these two cognitive tests.

The identification of the location and nature of brain changes related to the MMSE and SMMSE scores has received little attention to date [10–13]. Consistent with the present SMMSE findings, correlations between immediate and delayed free recalls were found with the GM volume of the left hippocampus in a smaller sample of 18 participants with MCI, 21 with AD and 11 CHI [13]. Additionally, correlations were reported between the MMSE score and the total GM volume [13], as well as the GM subvolumes of various brain regions including the parietal GM [13], the frontal GM [13], and the parahippocampal [12] and hippocampal subvolumes [11,12]. Of note, and consistent with our findings, the latter correlation was not mediated by the diagnostic category (i.e., MCI, AD, or CHI) [11]. Finally, Kovacevic et al. [10] interestingly revealed in 269 participants with MCI that baseline volumes of the hippocampus, amygdala, and temporal horn were independent predictors of MMSE changes over a 6-month follow-up. Consistent with these prior results, and without assuming any *a priori* regions of interest, we provided here further evidence that worse MMSE and SMMSE scores correlated with GM atrophy in the limbic system (i.e., a complex set of structures of the brain that lie on either side of the thalamus under the cerebrum) and in the middle temporal gyrus (located between the superior temporal gyrus and inferior temporal gyrus). Since the clinical expression of neurological damage is directly related to its location, our findings provide insight into the brain regions and associated functions that are actually probed by these tests. Specifically, the structures correlating with the MMSE score were i) the hippocampus, which plays a central role in the storage, consolidation and retrieval of explicit memories [22]; ii) the amygdala, which relates to perception and emotion including processing and storing memories of emotional events, or triggering affects and emotions from specific memories [23]; iii) the cingulate gyrus, which ends the Papez circuit supporting memory function [24]; iv) the parahippocampal gyrus, which is related to neurosensory structures of environment linking [25]; and v) the middle temporal gyrus, which has been connected with processes as different as recognition of known faces, and accessing word meaning while reading [26]. All together these results argue that the MMSE explores primarily learning and memory resources, but is also sensitive to motivation and perception of testing conditions, including the relationship to the examiner and the management of the environment, stress, and reading. These findings, including the lack of relationship between the MMSE and the subvolumes of the vast majority of brain structures, are consistent with the allegation that the MMSE should be considered more as a memory test than a composite test exploring the global cognitive performance of the whole brain in older adults without severe cognitive impairment [7,8]. However, our results also showed that the MMSE does not test solely memory, unlike the SMMSE. Indeed, we found that the SMMSE, which consists of learning, storing and returning a 3-word list [7], correlated with the volume of GM in the left hippocampus and left parahippocampal gyrus. These structures are both involved in memory recall [27] by binding together pieces of memory constructed not only by the hippocampus, but also by other regions of the brain, to be recalled at a later time [28]. According to Eichenbaum [27], the left hippocampus and parahippocampal area are critical for effectively combining the ‘what, ‘when,’ and ‘where’ qualities of each experience to construct the retrieved memory. Thus the very precise and unique location of the GM subvolume associated with performance on the SMMSE emphasizes the specificity of this test for evaluation of memory processing.

Some potential limitations of our study should be considered. First, the study cohort was restricted to Caucasian older outpatients followed in a memory clinic for a memory complaint therefore the results cannot be generalized to all older adults. Second, although we were able to control for important characteristics that could modify the correlation, residual potential confounders might still be present. Third, the SMMSE was built from the MMSE and not performed as a single test, which may induce variation in score. Other MMSE subscores were not available to determine how the atrophy pattern associated with each individual item corresponded to the global pattern. Fourth, VBM has been criticized because of segmentation and normalisation defects. Segmentation of brain into GM and WM is a major difficulty due to partial volume effects at the boundary between GM and WM, and because of mislabelling. We minimized these limitations by using SMP8 unified segmentation that is based on a generative model and thus performs better than previous versions. Moreover, major differences have been reported between registration algorithms. We tried to avoid this issue by using DARTEL, a fluid deformation algorithm capable of precisely realigning brain structures, which was one of the four highest-ranking registration methods in an evaluation of 14 non-linear deformation algorithms [29].

In conclusion, we found that worse MMSE and SMMSE scores correlated with GM atrophy mainly in the limbic system, independently of the diagnosis of CHI, MCI or AD. This finding highlights that the MMSE and SMMSE cognitive tests might be sensitive to changes in specific brain regions, and provides new insights into the interpretation of results from these two cognitive tests. Based on our results, it appears that both the MMSE and SMMSE explore learning and memory and that, although the SMMSE appears more specific to memory (left hippocampus), the MMSE may also be sensitive to motivation and perception of testing conditions (right amygdala). Further research is needed to corroborate this finding, and to determine whether specific mechanisms of atrophy can be identified by performance on the MMSE and SMMSE tests.

## References

[1] Folstein MF, Folstein SE, McHugh PR. "Mini-mental state". A practical method for grading the cognitive state of patients for the clinician. J Psychiatr Res. 1975;12: 189–198. 1202204 10.1016/0022-3956(75)90026-6

[2] Simard M. The Mini-Mental State Examination: strengths and weaknesses of a clinical instrument. Can Rev Alzheimers Dis Other Demen. 1998;2: 10–12.

[3] Nilsson FM. Mini Mental State Examination (MMSE)—probably one of the most cited papers in health science. Acta Psychiatr Scand. 2007;116: 156–157. 10.1111/j.1600-0447.2007.01037.x 17650282

[4] Creavin ST, Wisniewski S, Noel-Storr AH, Trevelyan CM, Hampton T, Rayment D, et al Mini-Mental State Examination (MMSE) for the detection of dementia in clinically unevaluated people aged 65 and over in community and primary care populations. Cochrane Database Syst Rev. 2016;1: CD011145 10.1002/14651858.CD011145.pub2 26760674 PMC8812342

[5] Mitchell AJ. A meta-analysis of the accuracy of the mini-mental state examination in the detection of dementia and mild cognitive impairment. J Psychiatr Res. 2009;43: 411–431. 10.1016/j.jpsychires.2008.04.014 18579155

[6] Brodaty H, Howarth GC, Mant A, Kurrle SE. General practice and dementia. A national survey of Australian GPs. Med J Aust.1994;160: 10–14. 8271977

[7] Haubois G, Annweiler C, Launay C, Fantino B, de Decker L, Allali G, et al Development of a short form of Mini-Mental State Examination for the screening of dementia in older adults with a memory complaint: a case control study. BMC Geriatr. 2011;11: 59 10.1186/1471-2318-11-59 21970520 PMC3203031

[8] Haubois G, de Decker L, Annweiler C, Launay C, Allali G, Herrmann FR, et al Derivation and validation of a Short Form of the Mini-Mental State Examination for the screening of dementia in older adults with a memory complaint. Eur J Neurol. 2013;20: 588–590. 10.1111/j.1468-1331.2012.03830.x 22913655

[9] Lazaro L, Marcos T, Pujol J, Valdes M. Cognitive assessment and diagnosis of dementia by CAMDEX in elderly general-hospital inpatients. Int J Geriatr Psychiatry. 1995;10: 603–609.

[10] Kovacevic S, Rafii MS, Brewer JB; Alzheimer's Disease Neuroimaging Initiative. High-throughput, fully automated volumetry for prediction of MMSE and CDR decline in mild cognitive impairment. Alzheimer Dis Assoc Disord. 2009;23: 139–145. 10.1097/WAD.0b013e318192e745 19474571 PMC2688740

[11] Morra JH, Tu Z, Apostolova LG, Green AE, Avedissian C, Madsen SK, et al Automated mapping of hippocampal atrophy in 1-year repeat MRI data from 490 subjects with Alzheimer's disease, mild cognitive impairment, and elderly controls. Neuroimage. 2009;45: S3–15. 10.1016/j.neuroimage.2008.10.043 19041724 PMC2733354

[12] Avila R, Ribeiz S, Duran FL, Arrais JP, Moscoso MA, Bezerra DM, et al Effect of temporal lobe structure volume on memory in elderly depressed patients. Neurobiol Aging. 2011;32: 1857–1867. 10.1016/j.neurobiolaging.2009.11.004 20031272

[13] Arlt S, Buchert R, Spies L, Eichenlaub M, Lehmbeck JT, Jahn H. Association between fully automated MRI-based volumetry of different brain regions and neuropsychological test performance in patients with amnestic mild cognitive impairment and Alzheimer's disease. Eur Arch Psychiatry Clin Neurosci. 2013;263: 335–344. 10.1007/s00406-012-0350-7 22940716

[14] Annweiler C, Montero-Odasso M, Bartha R, Drozd J, Hachinski V, Beauchet O. Association between gait variability and brain ventricle attributes: a brain mapping study. Exp Gerontol.2014;57: 256–263. 10.1016/j.exger.2014.06.015 24971908

[15] Dubois B, Sarazin M, Lehericy S, Chupin M, Tonelli I, Garner G, et al P2a-4 Etude Hippocampe: Evaluation de l’efficacité du donépézil versus placebo sur des marqueurs IRM et cliniques chez des patients présentant des troubles cognitifs légers. Revue Neurologique. 2009;165: 66–67.

[16] Ashburner J. A fast diffeomorphic image registration algorithm. Neuroimage.2007;38: 95–113. 10.1016/j.neuroimage.2007.07.007 17761438

[17] Good CD, Johnsrude IS, Ashburner J, Henson RN, Friston KJ, Frackowiak RS. A voxel-based morphometric study of ageing in 465 normal adult human brains. Neuroimage. 2001;14: 21–36. 10.1006/nimg.2001.0786 11525331

[18] American Psychiatric Association. Diagnostic and Statistical Manual of Mental Disorders 4th ed. Washington, DC: American Psychiatric Association; 1994.

[19] McKhann G, Drachman D, Folstein M, Katzman R, Price D, Stadlan EM. Clinical diagnosis of Alzheimer’s disease: report of the NINCDS-ADRDA Work Group under the auspices of Department of Health and Human Services Task Force on Alzheimer’s disease. Neurology.1984;34: 939–944. 6610841 10.1212/wnl.34.7.939

[20] Dubois B, Albert ML. Amnestic MCI or prodromal Alzheimer’s disease? Lancet Neurol. 2004;3: 246–248. 10.1016/S1474-4422(04)00710-0 15039037

[21] Eickhoff SB, Stephan KE, Mohlberg H, Grefkes C, Fink GR, Amunts K, et al A new SPM toolbox for combining probabilistic cytoarchitectonic maps and functional imaging data. Neuroimage. 2005;25: 1325–1335. 10.1016/j.neuroimage.2004.12.034 15850749

[22] Bartsch T. The Clinical Neurobiology of the Hippocampus: An integrative view Oxford: Oxford University Press; 2012.

[23] Sah P, Faber ES, Lopez De Armentia M, Power J. The amygdaloid complex: anatomy and physiology Physiol Rev. 2003;83: 803–834. 10.1152/physrev.00002.2003 12843409

[24] Vogt BA. Cingulate Neurobiology and Disease Oxford: Oxford University Press; 2009.

[25] Aguirre GK, Detre JA, Alsop DC, D'Esposito M. The parahippocampus subserves topographical learning in man. Cereb Cortex. 1996;6: 823–829. 8922339 10.1093/cercor/6.6.823

[26] Acheson DJ, Hagoort P. Stimulating the brain's language network: syntactic ambiguity resolution after TMS to the inferior frontal gyrus and middle temporal gyrus. J Cogn Neurosci. 2013;25: 1664–1677. 10.1162/jocn_a_00430 23767923

[27] Eichenbaum H. Comparative cognition, hippocampal function, and recollection. Comparative Cognition & Behavior Reviews. 2007;2: 47–66.

[28] Spreng RN, Mar RA. I remember you: A role for memory in social cognition and the functional neuroanatomy of their interaction. Brain Res. 2012;1428: 43–50. 10.1016/j.brainres.2010.12.024 21172325 PMC3085056

[29] Klein A, Andersson J, Ardekani BA, Ashburner J, Avants B, Chiang MC, et al Evaluation of 14 nonlinear deformation algorithms applied to human brain MRI registration. Neuroimage. 2009;46: 786–802. 10.1016/j.neuroimage.2008.12.037 19195496 PMC2747506

